# Neonatal Expanded Screening toward lysosomal storage disorders

**DOI:** 10.1186/1824-7288-41-S2-A11

**Published:** 2015-09-30

**Authors:** Alberto Burlina, Giulia Polo, Francesca Furlan, Monica Del Rizzo, Andrea Celato, Laura Giordano, Chiara Cazzorla

**Affiliations:** 1 Division of Inherited Metabolic Disorders, Department of Pediatrics, University Hospital, Padova, Italy

## 

Newborn Screening (NBS) is a public health program aimed at identifying treatable conditions in pre-symptomatic newborns to avoid premature mortality, morbidity and disability. The advent of tandem mass – spectrometry (MS/MS) has enabled the interrogation of multiple disorders using a single, multianalyte assay changing the origin scenario of one screening, one disease.

For example, even if a disorder was extremely rare, if it could have been detected and there were an effective intervention the minimal cost of adding it to a MS/MS panel might be cost effective.

Similarly, if one could add a disorder which there was no accepted effective treatment, it might be cost effective to add it based upon minimizing diagnostic testing to determine the cause of the phenotype and being able to counsel parents about their reproductive options.

This new based-technology prevention program, aimed at identifying an increasing number of conditions, fits for some lysosomal disorders (LSDs) such as Gaucher, Pompe, Fabry, MPSI, krabbe and Niemann-Pick diseases that have been proposed for inclusion in newborn expanded screening programs. In different Countries, pilot studies including all the above diseases or more selected disorders have already found the opportunity to validate the effectiveness of different methods, define the cut-offs for detection of the LSDs and alert the entire system of urgent referral, follow-up confirmation, treatment and screening program communication.

